# Transcriptomic Insights into the Molecular Responses of Red Imported Fire Ants (*Solenopsis invicta*) to Beta-Cypermethrin and *Cordyceps cicadae*

**DOI:** 10.3390/genes17010092

**Published:** 2026-01-17

**Authors:** Ruihang Cai, Xiaola Li, Yiqiu Chai, Zhe Liu, Yihu Pan, Yougao Liu

**Affiliations:** Wenzhou Key Laboratory of Resources and Development of Entomogenous Fungi, Zhejiang Institute of Subtropical Crops, Wenzhou 325005, China

**Keywords:** *Solenopsis invicta*, transcriptome, pesticide, entomopathogenic fungi, gene expression

## Abstract

Background: *Solenopsis invicta*, commonly known as the red imported fire ant (RIFA), is an important global invasive pest, and its management is challenging because of insecticide resistance and environmental problems. Methods: In this research, we applied transcriptomics to analyze the molecular responses of *S. invicta* worker ants exposed to different types of pesticides, beta-cypermethrin (BC) and the entomopathogenic fungus *Cordyceps cicadae* (CC), as well as to different concentrations of these pesticides. Results: A total of 2727 differentially expressed genes (DEGs) were identified across all samples. The number of DEGs in the BC treatment group was significantly higher than that in the CC treatment group (2520 vs. 433), and higher concentrations resulted in more DEGs (an increase of 47 in the BC group and 229 in the CC group). KEGG pathway analysis revealed that the DEGs were significantly enriched in lipid metabolism, carbohydrate metabolism, amino acid metabolism, signal transduction, and membrane transport. Immune-related gene analysis showed more general down-regulation (average FPKM value in BC 741.37 to 756.06 vs. CK 1914.42) of pathogen recognition genes (*PGRP-SC2*) under BC stress conditions, while CC treatment resulted in increases in expression of important immune effectors such as various serine proteases. Conclusions: Overall, this study provides useful insights into the molecular basis of responses to different pesticides in *S. invicta* and offers a basis to develop new approaches to control this pest.

## 1. Introduction

*Solenopsis invicta* Buren (Hymenoptera: Formicidae), the red imported fire ant (RIFA), is a highly invasive pest species indigenous to South America. After being introduced into non-native ecosystems, *S. invicta* primarily cause a decline in and local extinction of native arthropods, reptiles, birds, and small mammals through extreme competition and predation, thereby causing vast ecological damage [1,2]. It has also triggered widespread public health concerns in China [3,4,5]. In agriculture, *S. invicta* causes significant economic losses by directly damaging crops, by protecting phytophagous pests that produce honeydew, and by adversely affecting soil quality due to its mound-building activity [6]. In addition, *S. invicta* is highly aggressive and can inflict painful stings that lead to pustules, and for some affected individuals can cause potentially fatal anaphylactic reactions [7,8]. Its ability to adapt, to reproduce massively and its complex sociality all contribute to its rapid invasion and make it an important and persistent problem in regions occupied by *S. invicta* worldwide [9,10].

Although there are many control measures for ants and insects, rapid expansion and resistance to pesticides make pest management particularly challenging in the environment [11,12]. Chemical insecticides will continue to be the cornerstone of fire ant suppression, with beta-cypermethrin as a primary representative. However, the emergence of resistance, damage to non-target organisms, pollution of soil and water systems, and disruption of ecological balance have increasingly restricted the application of chemical insecticides [13,14,15,16,17,18]. Therefore, identifying and developing more sustainable and environmentally acceptable alternatives has become a key research area. Entomopathogenic fungi serve as a biological control strategy that is environmentally friendly, sustainable, and socio-ecologically compatible, holding significant application prospects for the control of *S. invicta*. However, biopesticides still have limitations in practical applications, such as strong environmental sensitivity and poor stability [19].

In recent years, the progression of high-throughput sequencing technology has provided important tools for systematically analyzing the gene expression regulation mechanisms of insects under pesticide stress [19,20,21,22]. In RIFA, chemical pesticides such as beta-cypermethrin and fipronil primarily upregulate genes related to detoxification metabolic pathways, such as Cytochrome P450 (CYP450), Glutathione S-transferase (GST), and Carboxylesterase (CarE), and affect energy and lipid metabolism pathways in response to toxicological stress [13]; other study has also found that after treatment with cycloxaprid, CYP450 and GST in *S. invicta* are briefly up-regulated in the early stage, followed by significant down-regulation at 24 and 48 h, leading to the gradual impairment of detoxification function [23]. In terms of biological stress, multiple studies have shown that entomopathogenic fungi such as *Beauveria bassiana* and *Metarhizium anisopliae* exhibit significant pathogenic and lethal effects on *S. invicta*. These fungi not only impact the behavior and physiological functions of the ants also achieve biological control by regulating the expression of host immune- and metabolism-related genes. Hassan et al. (2024) reported that after infection with *M. anisopliae*, the activities of peroxidase, GST, catalase, and CarE in *S. invicta* were significantly reduced, while antifungal activity increased and acetylcholinesterase activity decreased, indicating the suppression of its antioxidant and detoxification systems [24]. Through RNA-seq analysis, Wu et al. (2023) further confirmed the regulatory effect of fungal infection on the immune pathways of *S. invicta*, finding that *M. anisopliae* infection suppressed the expression of immune-related genes (*PGRP-SA*, *ModSP1*, *IMD*, etc.) at 12 and 24 h, while infection at 48 h may lead to up-regulation, reflecting the dynamic interplay between the host and pathogen [20]. Based on these findings, this study proposes the following hypothesis: chemical pesticide stress triggers broad metabolic and detoxification pathway responses in *S. invicta*, while specific immune pathways activated by fungal infections often exhibit dynamic regulation.

Although previous transcriptomic studies have separately elucidated the responses of *S. invicta* to various chemical insecticides (e.g., fipronil, indoxacarb, fluralaner and various neonicotinoids) or biological agents (e.g., *M. anisopliae*) [13,20,25,26,27], there has been a lack of direct comparative analysis under identical experimental conditions between a representative chemical neurotoxicant and an entomopathogenic fungus. *Cordyceps cicadae* is an entomopathogenic fungus in the family Claviciptaceae that has the potential to infect the nymphs of insects, and produce fruiting bodies on the exterior of their body surface [28]. *C. cicadae* has a wide range of hosts, showing high pathogenicity to various pests such as *Carposina sasakii* [29], *Plutella xylostella* [30], and *Spodoptera litura* [31,32]. This study aims to reveal the adaptive regulatory mechanisms of *S. invicta* worker ants in response to different types of stress at the molecular level by analyzing the transcriptome data of red fire ants after exposure to the chemical pesticide beta-cypermethrin and the entomogenous fungus *C. cicadae*. This study will provide a theoretical basis for identifying the targets of action between the two pesticides and developing new composite control strategies, thereby promoting the precise, efficient, and sustainable development of RIFA management techniques.

## 2. Materials and Methods

### 2.1. Insect Collection and Maintenance

*S. invicta* specimens utilized within the present research were gathered throughout Shanfu Town, Lucheng District, Wenzhou City in May 2025. Colonies were quickly transferred into a plastic container with a shovel and taken it back to the laboratory for cultivation. The container was treated with talc powder in advance to prevent ants from escaping [33]. The indoor culture temperature was 26 ± 1 °C, the humidity was 60 ± 5%, and ants were provided with a diet consisting of ham sausage and 10% sucrose solution. Before the experiment, ants were cultured in the laboratory for 2 weeks to ensure adaptation to the environment.

### 2.2. Pesticides and Treatment

Beta Cypermethrin Emulsion in Water (20%) was purchased from TIDEFO biotechnology Co., Ltd. (Nanning, China)., The *C. cicadae* 022017-9 strain was inoculated on PDA medium. After 10 days of cultivation at 24 °C in darkness, spores were collected, and a suspension of 1 × 10^7^ spores/mL was prepared with sterile water for use. The pathogenicity and virulence of this spore suspension, derived from the same strain 022017-9, have been previously validated through bioassays against *Rhopalosiphum padi* [34]. Four experimental groups were prepared: Beta cypermethrin 1 (BC1, diluted 2000 times), Beta cypermethrin 2 (BC2, diluted 1000 times), *C. cicadae* 1 (CC1, 10^6^ spores/mL) and *C. cicadae* 2 (CC2, 10^7^ spores/mL), with sterile water treatment as the control. 50 worker ants were used for biological testing each time. Before the bioassay, ants were deprived of food for 4 h and then fed different pesticide solutions. The control group was administered a comparable quantity of sterile water and 10% sucrose solution. After 24 h, workers were randomly collected from each sample and immediately transfer them to liquid nitrogen, then store them in a −80 °C freezer.

### 2.3. RNA Sequencing and Analysis

Full RNA isolation, sequencing library development and analysis transcriptome sequencing were conducted by Wuhan IGENEBOOK Biotechnology Co., Ltd. (Wuhan, China) (http://www.igenebook.com). The RNA extraction procedure was conducted as follows: approximately 60 mg of tissue was weighed and rapidly ground into powder under liquid nitrogen. An appropriate volume of TRIzol reagent was added (1 mL per 60 mg tissue), followed by vortex mixing and incubation at room temperature. After centrifugation, the aqueous phase was transferred and subjected to sequential extraction with chloroform and chloroform–isoamyl alcohol (24:1) to remove proteins and impurities. RNA was precipitated from the aqueous phase using isopropanol, collected by low-temperature centrifugation, washed twice with 75% ethanol, air-dried, and finally resuspended in nuclease-free water. RNA integrity and concentration were assessed by agarose gel electrophoresis and quantified using a Qubit fluorometer. Fundamentally, total RNA was procured via extraction method. All the RNA specimens were evaluated for their quality via the Qsep400 system (GUANGDING Biotechnology Co., Ltd., Changzhou, China). To construct RNA libraries with the MGIEasy mRNA Library Prep Kit (MGI Technology Co., Ltd. Shenzhen, China), 3 μg of total RNA was used [35]. The procedure included polyA-selected RNA extraction, RNA fragmentation, random hexamer primed reverse transcription, and 100 nt paired-end sequencing by DNBSEQ-T7 (MGI Technology Co., Ltd. Shenzhen, China).

### 2.4. Bioinformatics Analysis of RNA-Seq

The primer and subpar sequences were discarded via fastp (v0.21.0). Purified sequences were aligned onto the *S. invicta* reference sequences (GCA_016802725.1) by Hisat2 (version 2.1.0), permitting up to two mismatches [36]. Those genetic elements were matched with public protein databases; NR (RefSeq non-redundant proteins). Featurecount (v1.6.0) served as a means for mRNA quantification and standardization of gene activity levels in the context of FPKM (fragments per kilobase of transcript per million fragments mapped) [37]. Differentially expressed genes (DEGs) were identified with edgeR (v3.36.0) with a filter threshold of FDR < 0.05 and |log2FoldChange| > 1 [38], while all other parameters were set to default values. ClusterProfilerin R package (v4.2.2) [39] was employed to perform Gene Ontology (GO) [40] and Kyoto Encyclopedia of Genes and Genomes (KEGG) [41] enrichment analysis. The GO and KEGG enrichment analyses were calculated using hypergeometric distribution with a *p* value cutoff of 0.05.

### 2.5. RT-qPCR Validation

Four immune-related genes were randomly selected, and primers were designed using Primer Premier 5.0. The primers were validated using Oligo 7 and Primer-BLAST (https://www.ncbi.nlm.nih.gov/tools/primer-blast/index.cgi?LINK_LOC=BlastHome, accessed on 10 January 2026). All primers were synthesized by Tsingke Biotechnology Co., Ltd. (Beijing, China) (Table 1) and further screened by agarose gel electrophoresis. Actin was used as the reference gene. The qPCR reaction system (20 μL) using a fluorescent reagent kit (SYBR^®^Premix Ex TaqTM, TAKARA (Beijing, China)) consisted of: 2 × SYBR^®^ Premix Ex Taq II 10 μL, forward primer (200 nmol/L) 0.8 μL, reverse primer (200 nmol/L) 0.8 μL, template cDNA (5.0 ng/μL) 2 μL, and ddH_2_O 6.4 μL. The qPCR protocol was as follows: initial denaturation at 95 °C for 5 min, followed by 40 cycles of 95 °C for 10 s and 60 °C for 30 s.

## 3. Results

### 3.1. Sequencing, RNA-Seq Assembly, and Transcriptomic Analysis

RNA sequencing was performed on different treatment samples of *S. invicta*, with the base quality value Q30 in the raw data exceeding 96%. The BC1, BC2, CC1, CC2, and CK samples yielded 116,882,732, 123,828,396, 116,792,250, 113,310,552 and 124,830,758 raw reads, respectively. After quality control filtering, 116,862,220, 123,802,876, 116,765,722, 113,286,810, and 124,804,728 clean reads were obtained. The average numbers of clean reads aligned to the reference genome were 21,135,371, 26,651,051, 28,234,199, 25,757,154, and 27,602,814, respectively, with average alignment rates reaching 54.29%, 64.60%, 72.56%, 68.18%, and 66.37% (Table 2).

**Figure 1:**
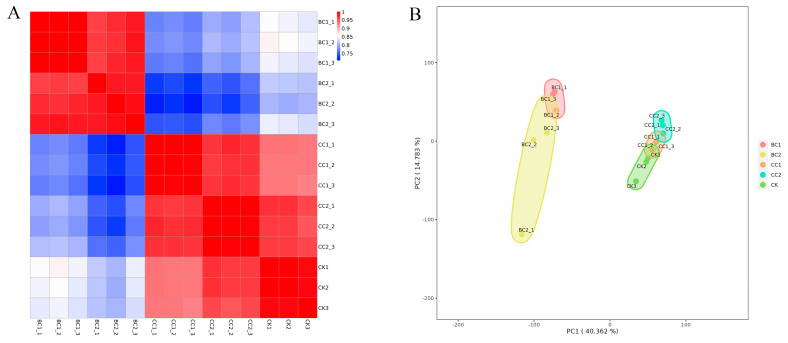
Principal component analysis between different treatments. (**A**): Heatmap of duplicate samples of different comparing groups of *S. invicta*; (**B**): PCA of transcriptomic data from different *S. invicta* comparison groups.

Following this, we assembled a transcriptional map comprising 14,471 genetic elements based on the RNA sequencing data from fifteen specimens (Figure 1A). Principal component analysis (PCA) of the transcriptome revealed distinct separation among the experimental groups (Figure 1B). The BC treatment group exhibited a significant divergence from both the CC and CK groups along the primary principal component (PC1). In contrast, the CC treatment group clustered much closer to the CK group, with the CC1 subgroup even showing partial overlap with CK, indicating a more similar transcriptomic profile.

### 3.2. Differentially Expressed Genes Analysis of S. invicta with Different Treatments

**Figure 2:**
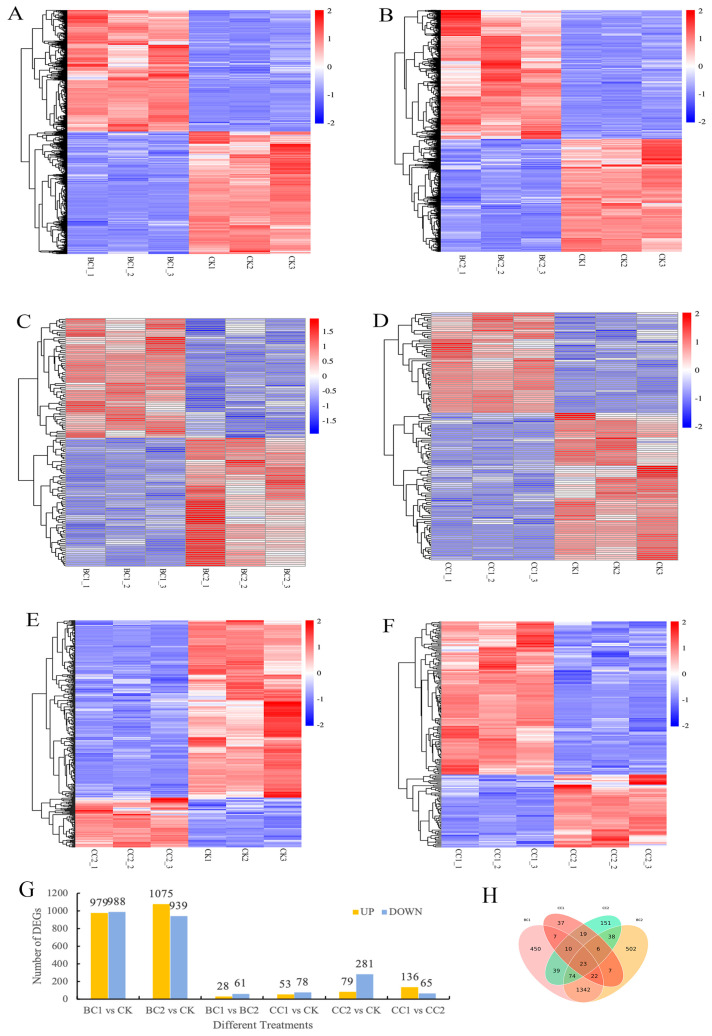
Transcriptomic analysis of *Solenopsis invicta* subjected to different chemical treatments. (**A**): DEGs of BC1 vs. CK; (**B**): DEGs of BC2 vs. CK; (**C**): DEGs of BC1 vs. BC2; (**D**): DEGs of CC1 vs. CK; (**E**): DEGs of CC2 vs. CK; (**F**): DEGs of CC1 vs. CC2; (**G**): the number of DEGs upregulated and downregulated; (**H**): the Venn diagram of DEGs.

Statistically significant DEGs were identified through pairwise comparisons of the control and treatment groups, with the criteria of |log2FC| > 1 and FDR < 0.05. Compared with CK, 2727 DEGs were generated in all treatment group samples (Figure 2). A total of 1967, 2014, 89, 131, 360 and 201 DEGs were identified in BC1 vs. CK, BC2 vs. CK, BC1 vs. BC2, CC1 vs. CK, CC2 vs. CK, CC1 vs. CC2 samples, respectively, employing a cutoff at |log2FC| > 1 and *p*-value < 0.05 (Figure 1). The Venn diagram (Figure 2H) revealed 1461 shared DEGs between BC1 and BC2, 58 shared DEGs between CC1 and CC2, 62 shared DEGs between BC1 and CC1, 141 shared DEGs between BC2 and CC2, and 23 shared DEGs across all treatments.

### 3.3. GO and KEGG Enrichment Analysis of Differentially Expressed Genes

**Figure 3:**
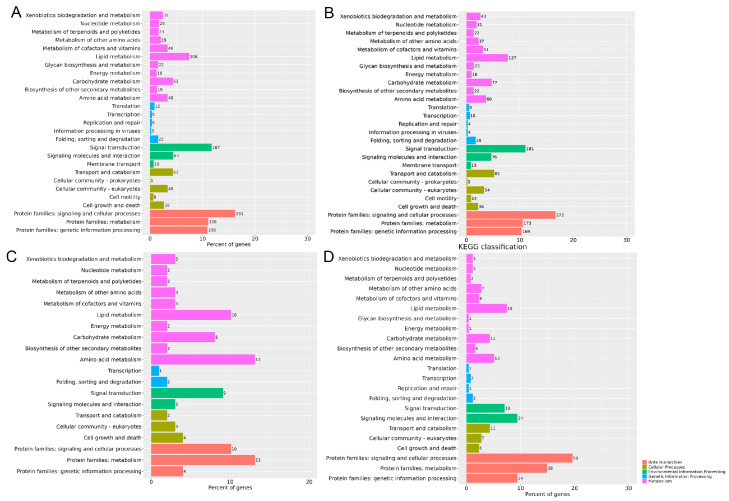
Analysis of significant enrichment of KEGG pathway in specific DEGs (*p*-value < 0.05, log2FC ≥ 10) under different chemical treatments. (**A**): BC1 treatment; (**B**): BC2 treatment; (**C**): CC1 treatment; (**D**): CC2 treatment.

**Figure 4:**
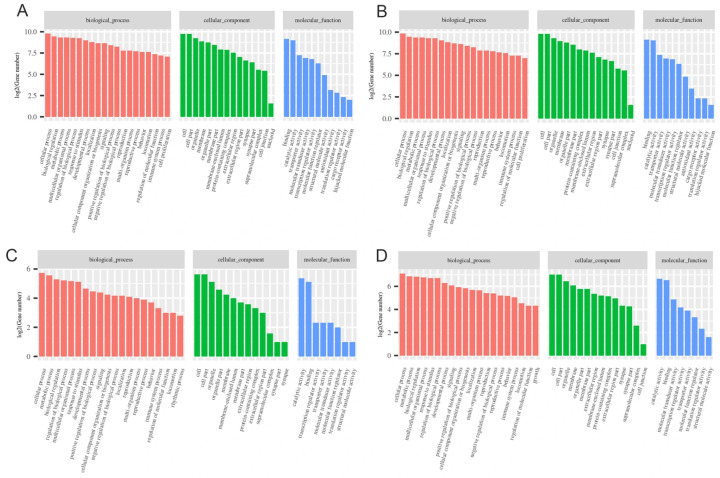
GO enrichment analysis of DEGs with different chemical treatments. (**A**): BC1 treatment; (**B**): BC2 treatment; (**C**): CC1 treatment; (**D**): CC2 treatment.

Specific unigenes (log2FC ≥ 10) for each condition were the subset of transcripts that were examined. Pathway and ontology analyses were used to confirm relevant transcripts (Figure 3 and Figure 4). Based on KEGG pathway analysis of four experimental groups (BC1, BC2, CC1, CC2) versus control (CK), distinct transcriptional response patterns emerged. The BC1 and BC2 (Figure 3A,B) groups significantly up-regulated genes involved in metabolic pathways, especially lipid metabolism (106, 127 genes, respectively), carbohydrate metabolism (62, 77 genes, respectively), and amino acid metabolism (48, 60 genes, respectively), as well as genes related to signal transduction (167, 181 genes, respectively) and membrane transport (10, 13 genes, respectively), indicating enhanced detoxification and neurotoxic stress response. In comparison, the CC1 and CC2 groups induced a wide range of transcriptomic changes. CC1 had a moderate upregulation in pathways involved with amino acid metabolism (13 genes); lipid metabolism (10 genes); and signal transduction (9 genes). In contrast, CC2 treatment had a stronger response in signaling molecules and interactions (24 genes), lipid metabolism (19 genes), and signal transduction (18 genes). In important metabolic pathways, specifically carbohydrate metabolism, lipid metabolism, along with amino acid metabolism, the count of altered genes in the CC2 cohort was significantly greater than that in the CC1 group. This shows that a higher concentration of spores had a more significant disruption of the basic metabolic activities of *S. invicta*. Furthermore, CC2 treatment drastically increased the number of genes in the signal transduction pathway, as well as protein families related to signaling and cellular processes. This indicates that treatment with spores at a higher concentration may activate more complex cellular defense and regulatory mechanisms. CC2 also showed a stronger impact on pathways such as cofactor and vitamin metabolism, and protein folding, sorting, and degradation, indicating that high-concentration treatment imposes higher requirements on cellular stress response and protein homeostasis maintenance. These results suggest that the transcriptional regulation of *S. invicta* by *C. cicadae* spores exhibits a concentration-dependent effect, with higher spore concentrations triggering more extensive metabolic reprogramming and activation of cellular signaling pathways.

Gene Ontology (GO) annotation was employed to elucidate the roles of differentially regulated genes (Figure 4). These DEGs were classified into three functional classifications: biological process (BP), molecular function (MF), and cellular component (CC). Among all treatment groups, the most important items are ‘biological processes: cellular processes’, ‘cellular components: cells’, and ‘cellular components: cell part’ (Figure 4A–D). The expression levels of DEGs in BC exhibited remarkably greater levels compared to those in the other treatment groups.

### 3.4. Analysis of Putative Genes Involved in the Immunity Mechanism

**Figure 5:**
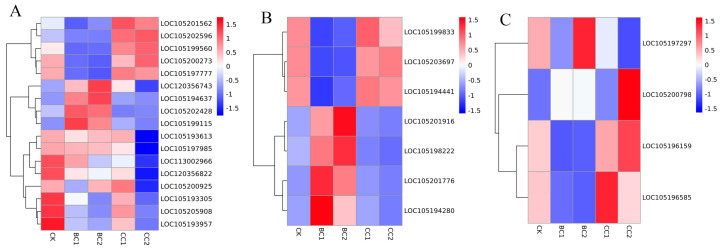
Heatmap of genes related to insecticide immunity. (**A**): Modulation-related genes; (**B**): signal transduction-related genes; (**C**): recognition-related genes.

Based on KEGG and NR annotations of the transcriptome data, systematic analysis identified 27 immune-related genes across different treatment groups. These genes were categorized into three functional classes, including recognition, signal transduction and modulation (Figure 5; Table S1). In the BC treatment groups, a general down-regulation of key immune recognition genes was observed. For instance, *PGRP-SC2* (*LOC105196159*) and *NPC2* (*LOC105197297*) showed reduced expression levels in BC groups compared to CK (average FPKM value = BC 741.37 to 756.06 vs. CK 1914.42), suggesting potential suppression of pathogen recognition capacity. In contrast, certain serine proteases were significantly up-regulated in BC groups, particularly under higher concentration (BC2), indicating a possible stress-induced proteolytic response. GST (*LOC105207856*) was also significantly induced, which is consistent with its function in detoxification processes. In the CC groups, expression levels of several immune-related genes remained nearer to CK or were moderately up-regulated. In particular, serine protease 3 (*LOC105202596*) was strongly induced in the CC groups, particularly CC2, suggesting it may be involved in antifungal defense mechanisms. Other genes such as *PGRP-SC2* (*LOC105196159*) and *serpin B* (*LOC105199560*) were induced (Average FPKM value = CC 2130.73 to 2670.06 vs. CK 1914.42, CC 481.54 to 528.67 vs. CK 354.00, respectively), in the CC groups as well, suggestive of the activation of humoral and regulatory immune pathways against the fungal infection.

### 3.5. qRT-PCR Validation of Immunity-Related Genes

**Figure 6:**
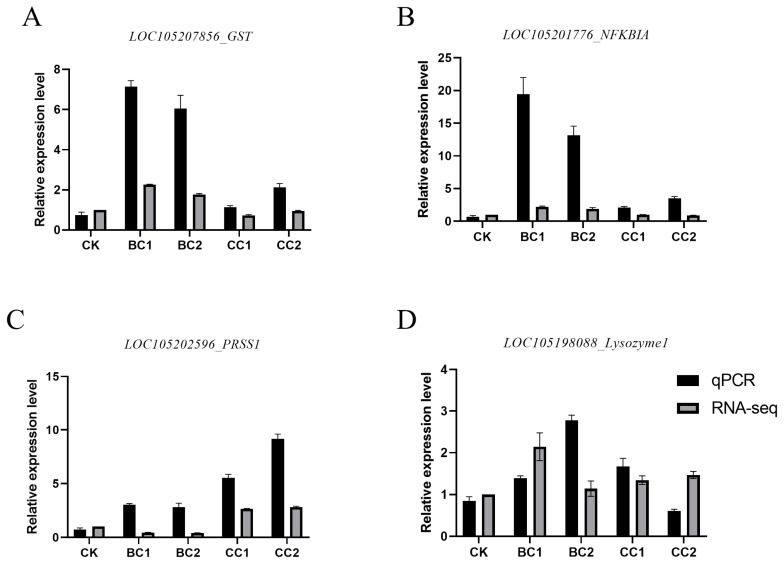
Comparison of the RNA-Seq and qRT-PCR results for different treatments. (**A**): *LOC105207856* gene; (**B**): *LOC105201776* gene; (**C**): *LOC105202596* gene; (**D**): *LOC105198088* gene.

In order to verify the accuracy of RNA-sequencing results, four immunity-related genes (*LOC105207856*, *LOC105201776*, *LOC105202596* and *LOC105198088* which were *GST*, *NFKBIA*, *PRSS1* and *Lyzosyme1*, respectively) were randomly selected from each sample for RT-qPCR analysis. The results showed that the expression levels of the four genes in each sample were consistent with the trend of expression changes in transcriptome sequencing data, further verifying the reliability of transcriptome sequencing data (Figure 6).

## 4. Discussion

Transcriptomic analysis in this study reveals distinct molecular response strategies in *S. invicta* to the chemical insecticide beta-cypermethrin and the entomopathogenic fungi *C. cicadae*. In the BC-treated groups, a significantly higher number of DEGs was induced compared to the CC treatment (2520 vs. 433), with higher concentrations further increasing DEG counts (2014 vs. 1967). These DEGs were primarily enriched in pathways related to lipid, carbohydrate and amino acid metabolism, as well as key detoxification enzyme families such as P450s, GSTs, and CarEs, indicating that beta-cypermethrin triggers widespread and intense activation of metabolic and detoxification pathways. This is consistent with the established neurotoxic mechanism of pyrethroid insecticides, which induce neuronal hyperexcitation and convulsions, leading to physiological stress and increased energy demands [42]. This “comprehensive mobilization” strategy represents a high-cost physiological investment aimed at rapidly metabolizing toxins and compensating for energy expenditure. Although effective in the short term, this “comprehensive mobilization” strategy imposes significant physiological costs and creates strong selection pressure for resistance. Indeed, Siddiqui et al. (2022) reported significant resistance evolution to fipronil (105.71-fold) and beta-cypermethrin (2.98-fold) in field populations of *S. invicta*, which was associated with enhanced activities of AChE, CarE, and GST [13]. This highlights the risk that reliance on potent neurotoxic agents like beta-cypermethrin, while eliciting strong transcriptomic responses, may accelerate the development of field resistance by selecting for individuals with pre-existing or induced enhanced detoxification capacity [43,44].

Furthermore, the response of *S. invicta* to *C. cicadae* infection was more modulated and dose-dependent, with only 433 DEGs identified. Furthermore, low-concentration spore treatment elicited only minor changes, suggesting that low doses of biopesticides may induce “immune priming” effects, potentially reducing subsequent control efficacy [45]. These findings provide a theoretical basis for “chemical–biological” sequential control strategies: first weakening the ant colony’s immune vigilance with chemical agents, followed by introducing entomopathogenic fungi to exploit their increased susceptibility. For example, the slow-acting, non-repellent neonicotinoid insecticide cycloxaprid disrupts the temporal regulation of lipid/energy metabolism and impairs olfactory perception genes in RIFA [23], while insect growth regulators can interfere with caste differentiation and social homeostasis [46]. These findings collectively suggest the development of a combinatorial strategy that integrates the multi-modal actions of chemical insecticides, entomopathogenic fungi, and insect growth regulators. Through synergistic or additive effects, this approach attacks the pest on multiple physiological fronts, reducing selection pressure on any single target while delaying the evolution of resistance [47]. Furthermore, compared to CK, all treatment groups shared 23 common DEGs, including glucose dehydrogenase (GLD, *LOC105203631*), odorant receptor-related genes (*LOC105198058*, *LOC113003171*, *LOC113004694*, and *LOC113006128*), and facilitated trehalose transporter Tret1 (*LOC105197969*). Previous studies have shown that GLD expressed in midgut cells of the diamondback moth is associated with insecticidal protein resistance, with its expression level reduced in Bt-resistant populations, suggesting that GLD may be involved in toxin metabolism or cellular protection [48]. In *S. invicta*, GLD expression was up-regulated in the BC treatment group but down-regulated in the CC treatment group. We speculate that GLD may play a role in detoxification or antioxidant protection under chemical stress, while its down-regulation during fungal infection could be a strategy to reallocate immune resources. Additionally, multiple odorant receptor-related genes were down-regulated across all treatment groups, indicating that both chemical and biopesticide treatments can interfere with the olfactory perception system of *S. invicta*, potentially affecting foraging, communication, or predator avoidance behaviors. Notably, compared to CK, only the high-concentration CC treatment induced the up-regulation of the trehalose transporter Tret1 gene, suggesting that under intense fungal stress, *S. invicta* may enhance trehalose transport to support energy supply and osmotic regulation required for immune responses.

## 5. Conclusions

In this study, transcriptomic analysis was conducted on worker ants of *S. invicta* exposed to different concentrations of the chemical insecticide beta-cypermethrin and the entomopathogenic fungus *C. cicadae*, systematically revealing the differential molecular response mechanisms of this pest to these two types of agents. The results showed that the number of DEGs induced by BC treatment (2520) was significantly higher than that induced by CC treatment (433), and higher concentrations further increased the number of DEGs. This indicates that *S. invicta* employs distinct strategies to cope with chemical and biological pesticides. When exposed to the potent insecticide cypermethrin, *S. invicta* activated extensive metabolic and detoxification processes, reflecting the energetic cost associated with processing neurotoxic substances. In contrast, in response to *C. cicadae* infection, it exhibited a more targeted, dose-dependent immune activation, suggesting its ability to “tune” its defense responses against fungal pathogens. In summary, this study elucidates the differential adaptation mechanisms of RIFA to chemical and biological stresses at the molecular level, providing a theoretical basis for developing integrated pest management strategies.

## Figures and Tables

**Table 1:** Primers used in this study.

Genes	Primer Name	Primer Sequences (5′ to 3′)
*Actin*	Actin-F	GCATGATCGGAAAGTGCG
	Actin-R	TTCAGCCACTTGACTGCG
*GST*	856-F	CGGCGTCAATAGGAGTGG
	856-R	AGGCTCGTTTTTTGGGGT
*NRKBIA*	776-F	GGGGGAGGCAGGTAGTTG
	776-R	CTGGCTCAGGGTCTCGGT
*PRSS1*	596-F	TTCTGGCTCTGTCATTCTCTC
	596-R	CTACTCTTACATCTTCGGCTTG
*Lyszyme1*	088-F	AGGCTGTATTTGCGAAGTTAG
	088-R	TCTCGTTGTTTAGAGTTGGTTT

**Table 2:** Statistical analysis of transcriptome sequencing data of *Solenopsis invicta* treated with different pesticides.

Sample	Raw Reads	Clean Reads	Mapped Reads (Mapping Ratio)	GC (%)	Q20 (%)	Q30 (%)
BC1_1	36,990,840	36,984,444	20,247,788 (54.75%)	38.61	99.35	97.22
BC1_2	43,369,054	43,361,724	23,327,463 (53.8%)	37.63	99.34	97.25
BC1_3	36,522,838	36,516,052	19,830,862 (54.31%)	38.82	99.34	97.19
BC2_1	41,610,378	41,603,650	25,560,163 (61.44%)	35.73	99.48	97.84
BC2_2	39,028,878	39,019,494	25,856,168 (66.26%)	39.01	99.31	97.09
BC2_3	43,189,140	43,179,732	28,536,821 (66.09%)	37.67	99.35	97.30
CC1_1	36,103,882	36,095,696	26,415,118 (73.18%)	38.95	99.27	96.88
CC1_2	42,632,520	42,623,122	30,866,339 (72.42%)	38.99	99.37	97.28
CC1_3	38,055,848	38,046,904	27,421,140 (72.07%)	38.63	99.37	97.26
CC2_1	36,659,382	36,651,402	25,045,137 (68.33%)	38.79	99.36	97.23
CC2_2	36,568,296	36,560,846	24,580,295 (67.23%)	38.53	99.32	97.12
CC2_3	40,082,874	40,074,562	27,646,031(68.99%)	39.66	99.38	97.32
CK1	37,519,836	37,510,904	25,060,975 (66.81%)	38.75	99.30	97.06
CK2	41,974,696	41,966,738	27,671,487 (65.94%)	38.05	99.34	97.24
CK3	45,336,226	45,327,086	30,075,982 (66.35%)	39.60	99.28	96.98

## Data Availability

A total of 15 RNA-seq raw reads from this study have been uploaded to Genbank with accession number PRJNA1364820.

## Supplementary Materials

The following supporting information can be downloaded at: https://www.mdpi.com/article/10.3390/genes17010092/s1, Table S1: Detoxification and immune related genes.

## References

[1] Khan S.A., Weemaels A.I., Liang M.R., Guénard B. (2025). Ecological and environmental impacts of the red imported fire ants (*Solenopsis invicta*) in mainland China, Hong Kong and Macau. Global Environ. Res..

[2] Allen C.R., Birge H.E., Slater J., Wiggers E. (2017). The invasive ant, *Solenopsis invicta*, reduces herpetofauna richness and abundance. Biol. Invasions.

[3] Song J.Y., Zhang H., Li M., Han W.H., Yin Y.X., Lei J.P. (2021). Prediction of spatiotemporal invasive risk of the red import fire ant, *Solenopsis invicta* (Hymenoptera: Formicidae), in China. Insects.

[4] Wang X.L., Qin Y.J., Xu Y.L., Feng X.D., Zhao S.Q., Lu Y.G., Li Z.H. (2023). Surveillance and invasive risk of the red imported fire ant, *Solenopsis invicta* Buren in China. Pest Manag. Sci..

[5] Korzukhin M.D., Porter S.D., Thompson L.C., Wiley S. (2001). Modeling temperature-dependent range limits for the fire ant *Solenopsis invicta* (Hymenoptera: Formicidae) in the United States. Environ. Entomol..

[6] Lafleur B., Hooper-Bùi L.M.H., Mumma E.P., Geaghan J.P. (2005). Soil fertility and plant growth in soils from pine forests and plantations: Effect of invasive red imported fire ants *Solenopsis invicta* (Buren). Pdeobiologia.

[7] Junior V.H., Larsson C.E. (2015). Anaphylaxis caused by stings from the *Solenopsis invicta*, lava-pés ant or red imported fire ant. An. Bras. Dermatol..

[8] Kemp S.F., DeShazo R.D., Moffitt J.E., Williams D.F., Buhner W.A. (2000). Expanding habitat of the imported fire ant (*Solenopsis invicta*): A public health concern. J. Allergy Clin. Immun..

[9] Xu Y.J., Lu Y.Y., Pan Z.P., Zeng L. (2009). Heat tolerance of the red Iimported fire ant, *Solenopsis invicta* (Hymenoptera: Formicidae) in mainland China. Sociobiology.

[10] Ross K.G. (1988). Differential reproduction in multiple-queen colonies of the fire ant *Solenopsis invicta* (Hymenoptera: Formicidae). Behav. Ecol. Sociobiol..

[11] Drees B.M., Calixto A.A., Nester P.R. (2013). Integrated pest management concepts for red imported fire ants *Solenopsis invicta* (Hymenoptera: Formicidae). Insect Sci..

[12] Zhang L., Wang L., Chen J., Zhang J.L., He Y.H., Lu Y.Y., Cai J.C., Chen X., Wen X.J., Xu Z.P. (2022). Toxicity, horizontal transfer, and physiological and behavioral effects of cycloxaprid against *Solenopsis invicta* (Hymenoptera: Formicidae). Pest Manag. Sci..

[13] Siddiqui J.A., Luo Y.Y., Sheikh U.A.A., Bamisile B.S., Khan M.M., Imran M., Haffeez M., Chani M.I., Lei N., Xu Y.J. (2022). Transcriptome analysis reveals differential effects of beta-cypermethrin and fipronil insecticides on detoxification mechanisms in *Solenopsis invicta*. Front. Physiol..

[14] Damalas C.A., Koutroubas S.D. (2018). Current status and recent developments in biopesticide use. Agriculture.

[15] Sakamoto H., Goka K. (2021). Acute toxicity of typical ant control agents to the red imported fire ant, *Solenopsis invicta* (Hymenoptera: Formicidae). Appl. Entomol. Zool..

[16] Wan N.F., Fu L.W., Dainese M., Kiaer L.P., Hu Y.Q., Xin F.F., Goulson D., Woodcock B.A., Vanbergen A.J., Spurgeon D.J. (2025). Pesticides have negative effects on non-target organisms. Nat. Commun..

[17] Chan K.H., Guénard B. (2020). Ecological and socio-economic impacts of the red import fire ant, *Solenopsis invicta* (Hymenoptera: Formicidae), on urban agricultural ecosystems. Urban Ecosyst..

[18] Barbieri R.F., Lester P.J., Miller A.S., Ryan K.G. (2013). A neurotoxic pesticide changes the outcome of aggressive interactions between native and invasive ants. Proc. R. Soc. B Biol. Sci..

[19] Mishra R., Chiu J.C., Hua G., Tawari N.R., Adang M.J., Sial A.A. (2018). High throughput sequencing reveals *Drosophila suzukii* responses to insecticides. Insects.

[20] Wu H.X., Xu Y.T., Zafar J., De Mandal S., Lin L.J., Lu Y.Y., Jin F.L., Pang R., Xu X.X. (2023). Transcriptomic analysis reveals the impact of the biopesticide *Metarhizium anisopliae* on the immune system of major workers in *Solenopsis invicta*. Insects.

[21] Shu B.S., Yu H.K., Li Y.N., Zhong H.X., Li X.L., Cao L., Lin J.T. (2021). Identification of azadirachtin responsive genes in *Spodoptera frugiperda* larvae based on RNA-seq. Pestic. Biochem. Phys..

[22] Wei N., Zhong Y.Z., Lin L.L., Xie M.H., Zhang G.L., Su W.H., Li C.R., Chen H.L. (2019). Transcriptome analysis and identification of insecticide tolerance-related genes after exposure to insecticide in *Sitobion avenae*. Genes.

[23] Du C., Jiang K., Xu Z., Wang L., Chen J., Wang C. (2023). Transcriptome and metabolome comprehensive analysis reveal the molecular basis of slow-action and non-repellency of cycloxaprid against an eusocial pest, *Solenopsis invicta*. Front. Physiol..

[24] Hassan A., Kang L.D., Zhang K.X., Wang L., Qin X.J., Fang G.B., Lu Y.Y., Huang Q.Y. (2024). Effect of entomopathogenic fungi on behavior and physiology of *Solenopsis invicta* (Hymenoptera, Formicidae). J. Econ. Entomol..

[25] Siddiqui J.A., Zhang Y., Luo Y., Bamisile B.S., Rehman N.U., Islam W., Qasim M., Jiang Q.J., Xu Y.J. (2022). Comprehensive detoxification mechanism assessment of red imported fire ant (*Solenopsis invicta*) against indoxacarb. Molecules.

[26] Xiong T., Ling S.Q., Liu J.L., Zeng X. (2020). Insecticidal and P450 mediate mechanism of fluralaner against Red Imported Fire Ant, *Solenopsis invicta* (Hymenoptera: Formicidae). Pestic. Biochem. Phys..

[27] Zhang B.Z., Kong F.C., Wang H.T., Gao X.W., Zeng X.N., Shi X.Y. (2016). Insecticide induction of O-demethylase activity and expression of cytochrome P450 genes in the red imported fire ant (*Solenopsis invicta* Buren). J. Integr. Agric..

[28] Li Z.Z., Hywel-Jones N.L., Luan F.G., Zhang S.L., Sun C.S., Chen Z.A., Li C.R., Tan Y.J., Dong J.F. (2020). Biodiversity of cordycipitoid fungi associated with Isaria cicadae I: Literature study. Mycosystema.

[29] Yaginuma K. (2002). *Paecilomyces cicadae* Samson isolated from soil and cicada, and its virulence to the peach fruit moth, *Carposina sasakii* Matsumura. Jpn. J. Appl. Entomol. Zool..

[30] Xu H.H., Hao Z.P., Wang L.F., Li S.J., Guo Y.R., Dang X.L. (2020). Suppression of transferrin expression enhances the susceptibility of *Plutella xylostella* to *Isaria cicadae*. Insects.

[31] Zhang S.L. (2020). Screening and Pathogenicity Test of *Cordyceps* sp. Strains Highly Virulent on *Spodoptera litura* (Fabricius). Master’s Thesis.

[32] Thanh D.D., Nishi O., Wasano N., Yasunaga-Aoki C. (2025). Identification of entomopathogenic fungus *Cordyceps cicadae* isolated from soil using common cutworm *Spodoptera litura* (Lepidoptera: Noctuidae) as bait and its high virulence comparable to generalist *Metarhizium anisopliae* complex. Fungal Biol..

[33] Ning D.D., Yang F., Xiao Q., Ran H., Xu Y.J. (2019). A simple and efficient method for preventing ant escape (Hymenoptera: Formicidae). Myrmecol. News.

[34] Liu Z., Liu Y.L., Chai Y.Q., Li X.L., Cai R.H., Liu Y.G. (2025). Identification of entomopathogenic fungus *Isaria cicadae* strain 022017-9 and its pathogenicity against the bird cherry-oat aphid *Rhopalosiphum padi*. J. Plant Prot..

[35] Chen S., Zhou Y., Chen Y., Gu J. (2018). Fastp: An ultra-fast all-in-one FASTQ preprocessor. Bioinformatics.

[36] Su W.L., Liu N., Mei L., Luo J., Zhu Y.J., Liang Z. (2019). Global transcriptomic profile analysis of genes involved in lignin biosynthesis and accumulation induced by boron deficiency in poplar roots. Biomolecules.

[37] Li B., Dewey C.N. (2011). RSEM: Accurate transcript quantification from RNA-Seq data with or without a reference genome. BMC Bioinform..

[38] Bakhtiarizadeh M.R., Salehi A., Alamouti A.A., Abdollahi-Arpanahi R., Salami S.A. (2019). Deep transcriptome analysis using RNA-Seq suggests novel insights into molecular aspects of fat-tail metabolism in sheep. Sci. Rep..

[39] Yu G., Wang L.G., Han Y., He Q.Y. (2012). ClusterProfiler: An R package for comparing biological themes among gene clusters. Omics.

[40] Ashburner M., Ball C.A., Blake J.A., Botstein D., Butler H., Cherry J.M., Davis A.P., Dolinski K., Dwight S.S., Eppig J.T. (2000). Gene ontology: Tool for the unification of biology. The gene ontology consortium. Nat. Genet..

[41] Kanehisa M., Goto S. (2000). KEGG: Kyoto encyclopedia of genes and genomes. Nucleic Acids Res..

[42] Soderlund D.M. (2012). Molecular mechanisms of pyrethroid insecticide neurotoxicity: Recent advances. Arch. Toxicol..

[43] Feyereisen R. (2011). Insect CYP genes and P450 enzymes. Insect Mol. Biol..

[44] Bass C., Jones C.M. (2018). Editorial overview: Pests and resistance: Resistance to pesticides in arthropod crop pests and disease vectors: Mechanisms, models and tools. Curr. Opin. Insect Sci..

[45] Contreras E., Rausell C., Real M.D. (2013). Proteome response of *Tribolium castaneum* Larvae to *Bacillus thuringiensis* toxin producing strains. PLoS ONE.

[46] Chouvenc T., Helmick E.E., Su N.Y. (2015). Hybridization of two major termite invaders as a consequence of human activity. PLoS ONE.

[47] Kliot A., Ghanim M. (2012). Fitness costs associated with insecticide resistance. Pest Manag. Sci..

[48] Guo Z.J., Zhu L.H., Cheng Z.Q., Dong L., Guo L., Bai Y., Wu Q.J., Wang S.L., Yang X., Wen X. (2024). A midgut transcriptional regulatory loop favors an insect host to withstand a bacterial pathogen. Innovation.

